# Does Dialogue Improve the Sustainable Employability of Low-Educated Employees? A Study Protocol for an Effect and Process Evaluation of “Healthy HR”

**DOI:** 10.3389/fpubh.2020.00446

**Published:** 2020-09-08

**Authors:** Emmelie Hazelzet, Hans Bosma, Angelique de Rijk, Inge Houkes

**Affiliations:** Department of Social Medicine, Faculty of Health, Medicine and Life Sciences, CAPHRI Care and Public Health Research Institute, Maastricht University, Maastricht, Netherlands

**Keywords:** low-educated employees, employer, dialogue, job control, sustainable employability, effect evaluation, process evaluation, protocol

## Abstract

**Background:** There is a need to develop sustainable employability (SE) interventions that are better aligned to the needs of low-educated employees. This group needs to get a voice in intervention development and implementation. In this study, a dialogue-based approach is proposed consisting of an online step-by-step support toolkit for employers, “Healthy Human Resources” (HHR). When intervening, this toolkit enables and stimulates employers to have a continuous dialogue with their low-educated employees. By improving the employees' job control, HHR is aimed at cost-beneficially improving SE. This paper describes the protocol of the evaluation study to evaluate the effectiveness and implementation process of HHR on the SE of low-educated employees.

**Methods:** The protocol of the evaluation study consists of: (1) an effect evaluation with a pretest-posttest design with a 1-year follow-up in five work organizations in the Netherlands deploying low-educated employees and with SE as the primary outcome and job control as the secondary outcome. The effect evaluation is expanded with a budget impact analysis; (2) a mixed-method process evaluation at 6 and 12 months after the start of HHR to evaluate the whole implementation process of HHR. This includes the experiences with HHR of various stakeholders, such as employees, human resource managers, and line managers.

**Discussion:** The effect evaluation will give insight into the effects of HHR on the SE of low-educated employees. The process evaluation will provide insight into the underlying mechanisms of the (in) effectiveness of HHR. By improving dialogue, we hypothesize that HHR, through enhancing job control, will strengthen the SE of low-educated employees. Also for helping with tackling the socioeconomic health gap, if proven effective, the implementation of HHR on a wider scale can be recommended.

## Introduction

Despite many attempts to reduce socioeconomic health differences, such differences remain large and persistent (1, 2). As, in the work domain, low-educated employees much more often prematurely leave the labor force due to health-related problems than their higher-educated counterparts (2–4), it is worrying that lower-educated employees are often difficult to reach in research and intervention efforts aimed at improving their situation (5, 6). Through absenteeism, presenteeism, and high staff turnover, this has substantial financial implications for employers too (7). Low-educated employees constitute a group that needs extra effort in this regard. Employees' sustainable employability (SE) has become top priority for employers, as they aim to foster employees' health and productivity in a sustainable way (8). The concept of SE is not one individual aspect, but rather an interaction between the employee and the organizational context. The workplace therefore is a good starting point to reach low-educated employees and improve their SE (8–10). This group, however, hardly participates in workplace health interventions (9, 11), and when they do participate, they tend to benefit to a lesser extent (12). In practice, many SE interventions are being developed without taking the perspective of the target group into account. Employers tend to buy ready-made health programs from (commercial) third parties, in which implementation takes place via a non-participatory top-down approach (13). Employees are often passive receivers in these programs (14, 15). Consequently, a mismatch occurs between these health programs and the needs and the world of daily experience of most low-educated employees. Therefore, low-educated employees need a different and more intensive approach than their higher-educated counterparts (16).

There is thus an urgent need to better align SE interventions to the needs of low-educated employees. To increase the effectiveness of these interventions, this group needs to have a say and needs to be actively involved in intervention development and implementation (6, 17, 18). Active involvement and participation in decision-making processes is expected to empower employees by increasing job control and autonomy; these in turn are expected to improve the employees' (mental) health and SE (19–21). Job control is an important determinant of employee well-being, particularly for low-educated employees who generally work in low control situations (20, 22, 23). When intervening, we expect that a profound dialogue between employees and the employer is crucial in increasing job control and SE among low-educated employees (24–26). Dialogue stands for an explanatory way of having a conversation in which all involved stakeholders experience a shared responsibility for the outcome of the dialogue (27). Instead of one-sided monologs or directives from the top, during dialogue, employees and representatives of the employer can think together and share experiences from different perspectives (25). When employers engage employees in dialogue, employees feel that their opinions count and that they are given a voice (28, 29). Previous studies found positive effects of improved work conditions through dialogue groups among high-educated physicians (28) and feeling heard and valued has been found to increase the self-esteem and self-efficacy of employees (19).

We propose a dialogue-based approach to stimulate active employee participation in the development and implementation of tailored SE interventions. We assume that this will contribute to a higher job control and SE of low-educated employees. Due to the participatory approach, including the dialogue component, employees get the opportunity to obtain more self-direction, experience more job control, which eventually will improve their health and SE. By lowering sickness absence, our approach will also be cost-beneficial for employers (7). We have therefore developed a free online support toolkit named - Healthy Human Resources' (HHR) aimed at improving SE of the low-educated employees. With the toolkit, employers (e.g., HR managers; supervisors), in dialogue with the low-educated employees, can develop and implement tailored SE interventions. As long as these are the outcome of a shared dialogue, the tailored SE interventions can vary widely regarding size and content and may, e.g., include compliments cards, job crafting, lifestyle interventions, or leadership training. The online toolkit HHR has already been developed, also in dialogue with several stakeholders, such as HR-managers, supervisors, and low-educated employees.

**Figure 1:**
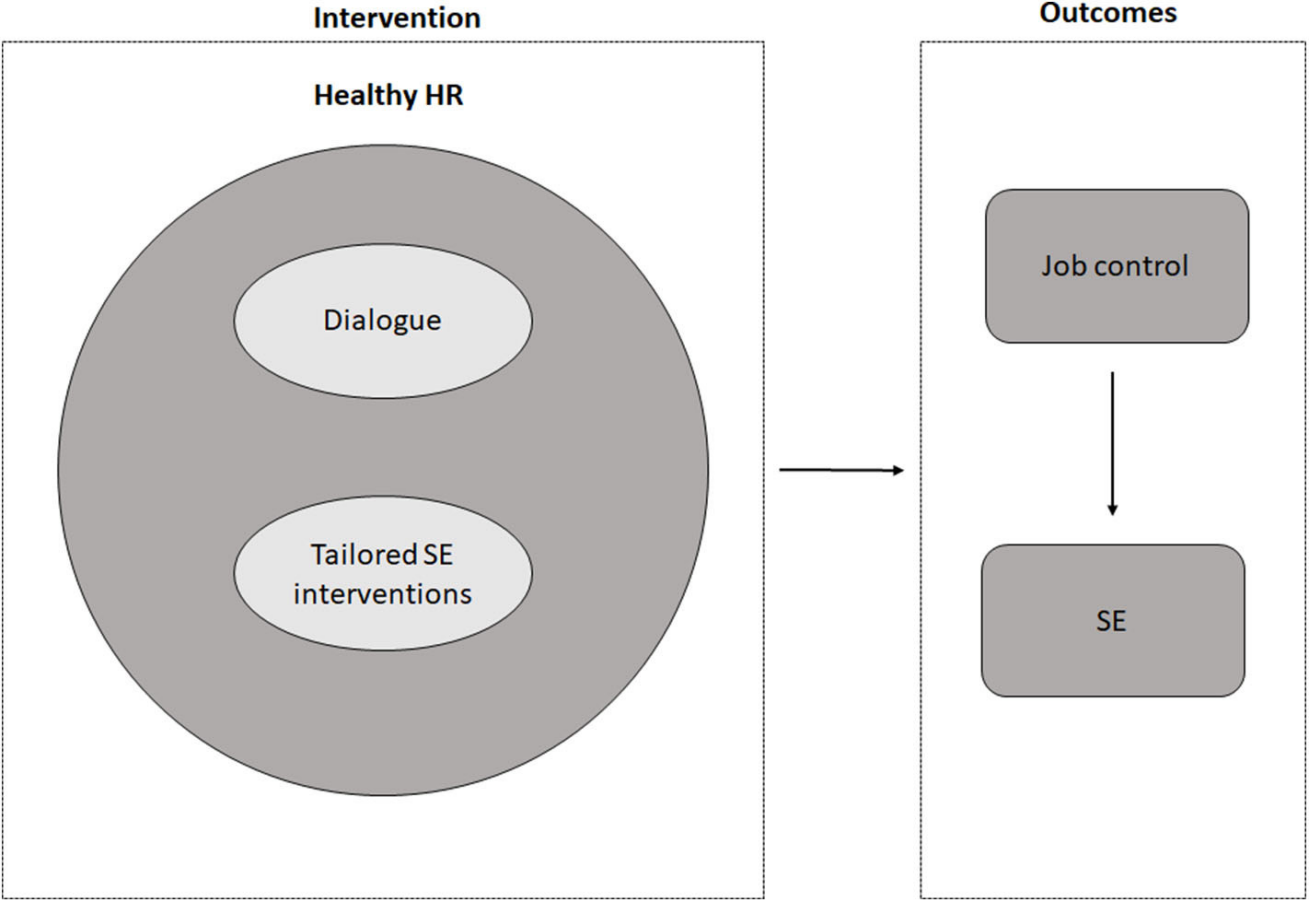
Conceptual model of HHR and expected outcomes.

This paper presents the study protocol of the evaluation study, evaluating the effect and the process of HHR. Particularly through increasing the low-educated employees' control at work, we hypothesize that the use of HHR in organizations, by integrating a dialogue-based approach, improves the SE of low-educated employees. We therefore also expect that employees who are more exposed to the dialogue integrated within HHR will experience more improvement in SE than employees who are less or not at all exposed to HHR (dose-response). The conceptual model of HHR is illustrated in Figure 1.

## Methods

The evaluation framework consists of a quantitative effect evaluation and an extensive mixed-method process evaluation. The aim of the effect evaluation is to investigate the effect of HHR on the SE of low-educated employees. The aim of the process evaluation is to assess the implementation process, the underlying mechanisms of the HHR's effectiveness or lack thereof (the how, what, why), and the HHR experiences of key stakeholders, such as the employees, HR manager and supervisors. The effect and process evaluation supplement each other.

### Intervention: Healthy HR

HHR is a web-based step-by-step support toolkit for HR managers and/or supervisors aimed at improving SE of low-educated employees. It supports HR managers and supervisors by developing and implementing their own tailored SE interventions by – from the start – involving their low-educated employees via dialogue. This online toolkit is presented on the “Healthy Human Resources” website (www.gezondhr.nl) (in Dutch). It consists of different steps, tasks, and dialogue-based tools for use within a team or department of the participating organizations. Within HHR eight steps are presented: step (1) Prepare together; step (2) Measuring is knowing; step (3) Our problems; step (4) Our solutions; step (5) Action plan; step (6) Let's start; step (7) Evaluation, and step (8) Along the way: obstacles in the process. Each step, is represented by several underlying tasks (e.g., brainstorming; prioritizing; communicating) and every task contains one or more supportive tools. Tools can be questionnaires, working forms, checklists, communication tips and information, external links, or a library with simple solutions and evidence-based interventions. Every task and tool facilitates a certain degree of employee participation and dialogue. The main outline of the steps, tasks and tools are presented in Appendix A. Organizations can select the tools which best fit to their context and their employees' situation, thereby developing a tailor-made toolkit for the needs assessment (HHR step 1–4) and developing and implementing their own tailored SE interventions (HHR step 5–7). The development of HHR is based on the Intervention Mapping approach (IM) (30). As IM is a rather detailed and time-consuming approach (30, 31), we decided to use an adapted version of the IM within HHR as well; this will make HHR more feasible for employers to put into practice (32, 33). The HR manager and/or supervisor will facilitate HHR themselves, without any external consultancy. We developed HHR in such a way, that it is a self-led intervention. It will be delivered in the participating organization, likely during working hours. HR manager and supervisors are able to decide by themselves how much time they spend on HHR and how they are going to integrate HHR in the daily business. However, a rule of thumb is provided within the toolkit by the researchers. Nevertheless, we expect when using HHR more frequent and more intense, employees will be more exposed and will experience more improvement on SE as mentioned before. A detailed description about the development and content of HHR will be published elsewhere (34).

### Effect Evaluation

The effect evaluation will be a quantitative study with a pretest-posttest design with a 1-year follow-up within each participating organization (T2). The employees' SE will be compared between prior to and after the HHR intervention. We will also examine whether the SE improves more, if employees are more exposed to HHR. Additionally, a budget impact analysis (BIA) will be performed to gain more insight into whether HHR is financially affordable and beneficial for employers deploying low-educated employees. The primary aim of the effect evaluation is to investigate the effectiveness of HHR on the SE of low-educated employees. The main research question is:

- What is the effect of HHR on the SE of low-educated employees?

#### Study Sample and Sample Size

Five Dutch work organizations (a manufacturing company, a meat processing company, a cleaning company, a warehouse and a governmental institution) participated in the development of HHR. These organizations will also implement HHR and participate in the effect evaluation. Employees with lower educational levels varying from no education to secondary vocational education [coded according to the 2011 International Standard Classification of Education (ISCED-11)] will be included in HHR and the effect evaluation. In this study, we will focus on employees with lower educational levels, particularly those employees who perform low-skilled jobs within certain departments of an organization.

A power calculation was performed to determine the sample size. Based on the mean difference in SE of 0.25 (theoretical range 1 to 5) that was found between high and low-educated employees in a previous study (35), we expect SE differences between high and low-educated employees to decrease with 0.25. As the uptake and output of HHR is organization-specific, we aim to study the SE improvement in each organization separately, but we will also pool the data to examine the overall effect. With a power of 80% and a significance level of 5%, the required sample size is a minimum of 126 employees per organization (36), which implies an overall sample size of 630 employees. We expect a varied non-response and dropout rate per organization. The gross number of employees varies between 40 and 1,200 per organization. For participating organizations with insufficient power, data will be pooled.

#### Data Collection

**Figure 2:**
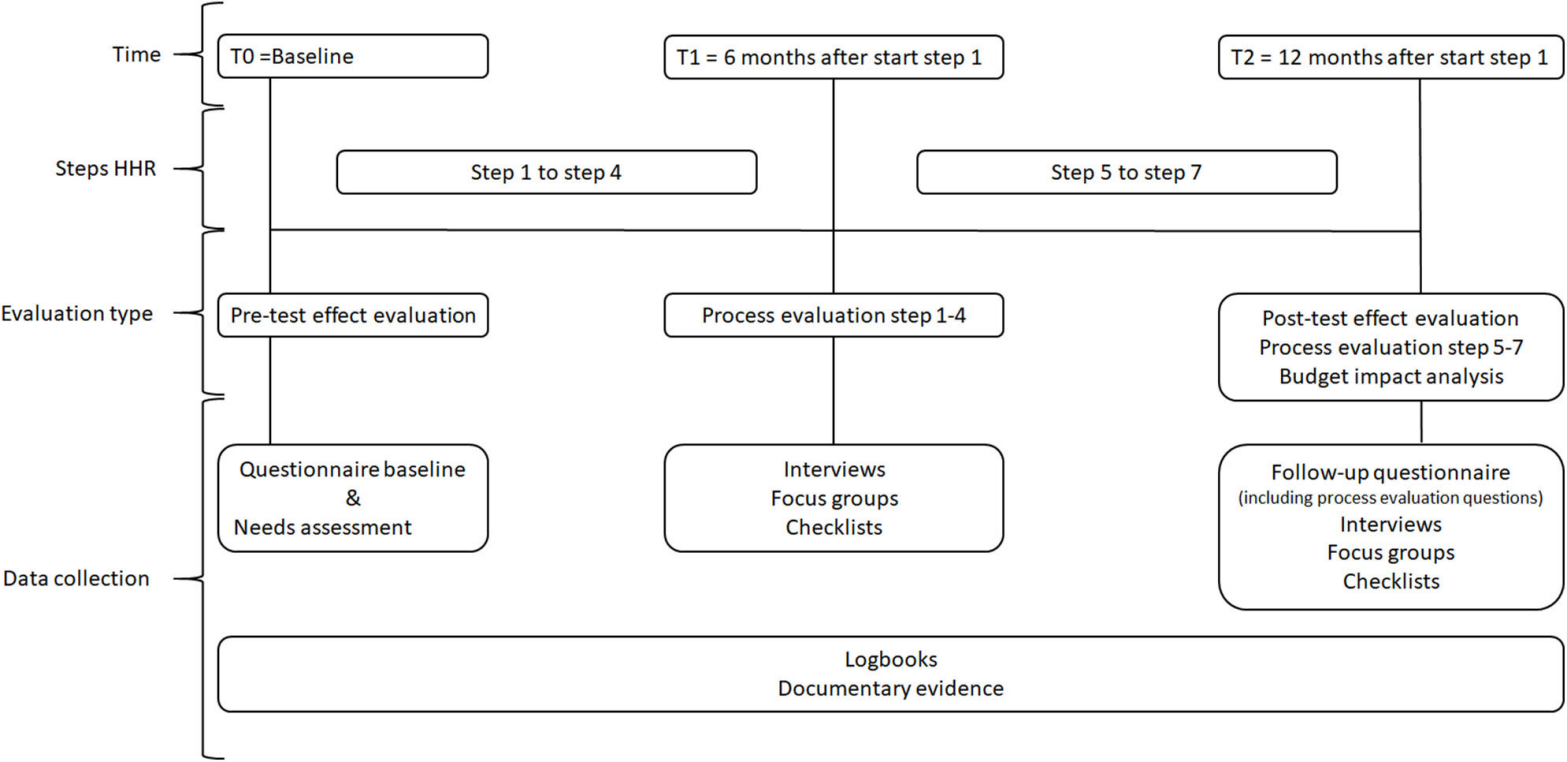
Overview evaluation moments and data collection.

Data for the effect evaluation of HHR will rely upon quantitative data from similar questionnaires at two time points: baseline (T0) and follow up (T2, 12 months after the start of step 1) (Figure 2). The baseline questionnaire (T0) will also be used as the needs assessment instrument in step 2 of HHR. The questionnaire for the needs assessment and effect evaluation is adapted and based on the existing Maastricht Instrument of Sustainable Employability (MAISE) (35). The MAISE has been developed for measuring SE from an employees' perspective. The MAISE has been validated among employees with (on average) intermediate and higher educational levels. For use among a sample of low-educated employees and the purpose of serving as a needs assessment, the MAISE and other (self-developed) subscales, such as job control, self-efficacy and lifestyle have been adjusted, to better fit with the language and way of thinking of low-educated employees. It is our hope that this adaptation improves the reach and the validity and reliability of our questionnaires. For instance, the use of existing job control scales from existing questionnaires were still too difficult to understand by the employees when discussing these items together with them. For the effect evaluation, additional, well-validated measures were also used (e.g., vitality).

##### Primary outcomes

Sustainable employability (SE) will be the primary outcome of the effect evaluation and can be considered as a distal outcome measure. The level of SE is measured by means of two scales, productivity and health, from the Maastricht Instrument of Sustainable Employability (MAISE) (35). SE measurement will be complemented by several proxies of SE:

Vitality will be measured by means of the subscale vitality of the Dutch version of the Utrecht Work Engagement Scale (UWES) (5 items) (37). The response scale ranged from 1 (never) to 7 (always/every day). A global measure of work engagement will be used as well, measured by means of the shortened Dutch version of the Utrecht Work Engagement Scale (UWES-3). This short version of UWES-9 is proven to be reliable and valid (38). Self-perceived health will be measured using a single item: “In general, what would you say about your health?” with five response options: excellent; very good; good; fair; and poor. For sickness absence, self-reported sickness absence will be measured by using a single item: “In the past 12 months, how many days were you sick-listed?” and registered sickness absence data will be drawn from the registers of the organizations. The sickness absence percentages will be obtained per participating department of each organization before the start at T0 and after 12 months (T2).

##### Secondary outcome

Job control will be the secondary outcome of the study and will be measured by means of a self-developed scale consisting of 5 items. The items are inspired by existing lists, such as the Dutch Questionnaire on the Experience and Evaluation of Work and the Maastricht Autonomy questionnaire (39, 40). The formulation of the items was aligned to the linguistic usage and preferences of the low-educated employees. The response scale ranged from 1 (never) to 5 (always). An example item is: “I have a say in what happens on my job.” Validity and reliability of this scale will be analyzed.

##### Other outcomes

We included several additional proximal outcomes which can be used to measure potential effects of the tailored SE interventions per organization: self-efficacy, lifestyle, social climate, social support, organization of work, adapted work possibilities, and communication and collaboration. Self-efficacy will be measured by means of the general self-efficacy scale (GSES-12) using the subscale effort (5 items) (41). Lifestyle will be measured according to the five behaviors: physical activity, smoking, alcohol use, consumption of fruit or vegetables and quality of sleep (42–44). These five lifestyle behaviors provided a so-called “optimal lifestyle index.” Each behavior scored “1” when the norm is met (and “0” when not met). A sum score will be computed of all five behaviors to create an optimal lifestyle index (43). The variables social climate (4 items), social support (3 items), organization of work (9 items), adapted work possibilities (4 items), and communication and collaboration (5 items) will be measured by means of self-developed scales. Validity and reliability of these scales will be analyzed.

Information on covariates (gender, age, type of contract (e.g., permanent or flex), level of education, ethnicity, shift work) will be also collected. Finally, to examine whether the SE improves more when employees are more intensely exposed to HHR (dose-response), the process indicator dose-received will be included in the follow-up questionnaire (T2). Dose-received will be measured by means of a self-developed continuous scale at employee and organizational level (see also process evaluation). Employees will be asked to what extent they actively aware and participated in HHR.

#### Data Analysis

Descriptive statistics will be used to analyze background characteristics. Differences over time (T0-T2) on the primary and secondary outcomes will be analyzed by means of paired *t*-tests of mean differences, chi square tests and regression analyses. The dose-received variable will be used to test the correlation between the dose and change in the primary outcome SE. Subgroup analyses (e.g., gender; education; type of contract) will be performed to examine specifically heightened or lowered improvements in SE in subgroups. Multilevel analyses are used to examine the association between the level of HHR implementation on the company level (level 2) and the improvement in SE (level 1). If multilevel analyses appear not to be feasible, other ways of taking account of the nested design will be considered. Finally, when there is a need for pooling (one organization has only 40 employees in total), multilevel is similarly considered (when pooling). Analyses will be performed using SPSS version 26.

#### Budget Impact Analysis

We will perform a budget impact analysis (BIA) from the employer perspective. The main aim of the BIA is to assess whether the implementation of HHR is financially affordable for the employer (e.g., time; implementation costs of HHR; additional cost for HHR) and show the budget impact of HHR. Generally, employers have interest in maintaining a healthy and productive workforce and, thus, they may be able to offset decreased sickness absence gains against the costs. Data will be collected on the direct costs of specific resources needed to implement HHR (e.g., staff, expertise, supplies, equipment, working time) by means of interviews. The estimation of the time spent gathered in interviews will be supplemented with data from the logbooks of the employers and researchers. The time spent will be translated to costs by multiplying number of hours with the average hour salary of for the group of employees involved in HHR. We ensure that the report on both costs and benefits will be simultaneously available for employers and HR managers.

### Process Evaluation

The aim of the process evaluation is to evaluate, in each participating organization, the implementation process and the underlying mechanisms of the HHR's effectiveness or lack thereof (the how, what, why), and the experiences of key stakeholders with HHR. These key stakeholders might influence the implementation throughout the process in various ways and therefore the outcomes. The process evaluation will have a mixed-method design (45) and will be utilized to interpret and understand the outcomes of the effect evaluation (46, 47). The study population of the quantitative process evaluation (follow-up questionnaire T2) equals that of the effect evaluation (the employees). The study population of the qualitative process evaluation includes various stakeholders (i.e., employees, supervisors, and HR managers) at different levels of the organizations. We will examine the key process indicators suggested by Linnan and Steckler presented in Table 1 (48). Because the organizational context can hinder or facilitate the implementation process and outcomes, we will examine both omnibus context (e.g., general context) and discrete context (e.g., specific events during HHR) in this process evaluation (46, 47, 49). In the qualitative parts of the process evaluation, we will generally follow the principles of responsive evaluation, which is well in line with the participative and dialogue-based approach of this study (50). This participative evaluation method explicitly includes the intervention and connects the different perspectives of stakeholders in order to obtain a more complete picture.

**Table 1:** Process indicators, stakeholders' level, operationalization and data collection method.

**Process indicators and definition**	**Stakeholder level**	**Operationalization**	**Data collection method**
*Context* The contextual factors (omnibus; discrete) and history (i.e., barriers, facilitators) that affect HHR implementation or outcomes	Employer Employees	Description of barriers Description of facilitators	Documentary evidence (T0–T2) Logbook (T0–T2) Focus groups (T1; T2) Semi-structured Interviews (T1;T2)
*Recruitment* Procedures used to approach and attract employees	Employer Employees	Description of approaches	Logbook (T0–T2) Focus group (T1; T2)
*Reach* Percentage of departments and employees participating in HHR	Employees	Characteristics of departments Characteristics of employees Percentage of employees, participated Drop-out and reasons	Baseline questionnaire and follow-up questionnaire (T0; T2) Logbook (T0–T2) Focus groups (T1; T2) Semi structured Interviews (T1; T2) Checklist (T1; T2)
*Dose delivered* The extent to which HHR or components actually was delivered according to the intervention plan	Employer Employees	Dose delivered items (yes/no)	Logbook (T0–T2) Questionnaire at follow-up (T2) Focus groups (T1; T2) Semi structured Interviews (T1; T2) Checklist (T1; T2)
*Dose received* The extent to which employees actively aware and participated in HHR	Employees	Dose-response Participation rate HHR	Questionnaire at follow-up (T2) Focus groups (T1;T2) Semi structured Interviews (T1;T2)
*Fidelity* The extent to which HHR was delivered as intended	Employer Employees	Statements (yes/no) Reasons	Logbook (T0–T2) Questionnaire at follow-up (T2) Focus groups (T1; T2) Semi structured Interviews (T1; T2)
*Satisfaction* Employees and employer satisfaction about HHR	Employer Employees	Satisfaction rate (scale 0–10) Experiences of employees and employers	Logbook (T0–T2) Questionnaire at follow-up (T2) Focus groups (T1; T2) Semi structured Interviews (T1; T2)

The research questions for the process evaluation are:

- How and to what extent has HHR been implemented in the participating organizations, taking into account the key process indicators?
- What are the experienced changes and the perspectives of the key stakeholders with HHR?

#### Data Collection and Analysis

Data will be collected throughout the entire process (T0-T2), at 6 months (T1), and at 12 months (T2) after the start of step 1 of HHR (Figure 2). In order to gain multiple perspectives and assure data validity, data source triangulation will be applied (51). At T2, the follow-up questionnaire of the effect evaluation will be extended with quantitative process evaluations questions covering the key process indicators: Reach, dose delivered, dose received, fidelity and satisfaction. These quantitative data will be analyzed by means of descriptive statistics. Data on the process indicators will be collected by means of different methods and at different stakeholder's levels within the organization (Table 1). Throughout the process (T0-T2), employers have the opportunity to give feedback by means of a feedback function built within HHR. Employers will keep track of the progress, number of meetings, time investment, participants, special remarks and events by means of a logbook and will be called monthly by the researchers. The researchers will also keep a logbook to document events and to keep documentary evidence for each participating organization. At T1, we will collect qualitative data about the experiences of employees and employers with steps 1–4 of HHR. At T2, we will collect qualitative data about the experiences of employees and employers with steps 5–7 of HHR (Figure 2). For both T1 and T2, focus groups and individual semi-structured interviews with the key stakeholders and other third parties (e.g., policy makers; communication staff) involved in the process will be conducted. These individual interviews and focus groups are complementary to each other (52). The topic lists for the focus groups and individual semi-structured interviews will be based on the process indicators and will include open-ended questions about HHR, the dialogue-based approach, experiences of stakeholders with HHR, and experienced changes. All focus groups and individual interviews will be digitally recorded and qualitative data will be analyzed thematically via a qualitative data analysis software program (e.g., NVivo).

## Discussion

This paper presents the protocol for the effect and process evaluation of the intervention HHR. HHR is a web-based support toolkit for employers based on dialogue and aimed at improving the SE of low-educated employees. We hypothesize that - through increasing job control - employees who are more exposed to HHR will experience better SE than employees who are less or not exposed to HHR.

### Strengths of the Protocol

This evaluation study provides insight into the effect and implementation process of HHR, including the underlying mechanisms that shapes the outcomes. Data triangulation using different quantitative and qualitative methods and data sources will be applied to assure the validity of this research. We expect that HHR as a whole will show positive effects on the SE of low-educated employees, regardless which organization or the effects of the tailored SE interventions developed per organization and the way we organized the process evaluation supports finding explanations for possible lack of effects. Furthermore, an economic perspective from the employer is also taken into account in a form of a BIA. The BIA will address the affordability of HHR and, together with the report on the benefits and gains of the intervention, will help employers to decide whether they want to invest in HHR.

The extensive process evaluation, including different time points and data collection methods, will be a strength to better understand the underlying mechanisms of HHR, experienced changes and how dialogue and job control is experienced by different key stakeholders over time. Furthermore, the process evaluation at T2 allows to gain insight into the specific tailored SE interventions in each organization and their related perceived effectiveness next to the experience of HHR as a whole. Finally, we conduct the evaluation study in five different sectors and settings, which will increase the generalizability of our results.

### Methodological Challenges

Despite this extensive study design, several methodological challenges can be pointed out. First, HHR is a generic toolkit and organizations will work with the same steps, tasks and tools. However, the way HHR will be implemented, including the use of the tools will differ per organization. Employers are free to choose those tools which best fits their situation and their specific SE problems. This might lead to differences in effects and processes across the organizations. Therefore, it is important to perform subgroup analyses. Second, the participating organizations appeared to be unable to allocate a control group, because of time limits and other concerns within organizations. The lack of a control group is a well-known issue within research of organizations; this unfortunately leads to less robust evidence about what is effective in terms of SE interventions in the workplace (53). Hence, due to the lack of the control group it is important to study the uptake of HHR and profoundly assess whether there is a dose-response relationship. Third, the setting and context within participating organizations will be a challenge, due to constant changes (e.g., dismissing/attracting flex workers; changing role/attitude of key stakeholders). Fourth, it may vary per organization how much time the HR managers and the wider management will allow to spend by their employees, e.g., for filling in questionnaires (including the needs assessment) and to work with HHR. This is also related to the level of commitment and support of the higher management. These changes might affect the results and will therefore be well-documented throughout the process and assessed during the process evaluation moments (e.g., being dismissed clearly is a low control experience for the employee).

Despite these methodological challenges, it is important to conduct evaluation studies in natural settings of organizations and among low-educated employees in particular. Their voices need to be heard, also in research. If HHR is proven to be effective, HHR for and with this vulnerable group will be a valuable support toolkit, which can be applied on a wider scale. HHR is thereby expected to contribute to tackling the socioeconomic health gap.

## Ethics Statement

The Medical Ethical Committee (METC) of the academic hospital (MUMC) in Maastricht confirmed that the Medical Research Involving Human Subjects Act (WMO) does not apply to this study and that an official approval of this study by the committee is not required (METC 2017-0311). All participants in the effect and process evaluation will be asked to sign an informed consent form when they start their participation in the study.

## Author Contributions

EH wrote the original draft. EH, AR, HB, and IH reviewed, revised, and edited several earlier versions of this paper. All authors read and approved the final version of the manuscript and contributed to finalizing the design and protocol for the study.

## Conflict of Interest

The authors declare that the research was conducted in the absence of any commercial or financial relationships that could be construed as a potential conflict of interest.
