# Supplementary material for: Does Dialogue Improve the Sustainable Employability of Low-Educated Employees? A Study Protocol for an Effect and Process Evaluation of “Healthy HR”
Source: Front Public Health. 2020 Sep 8;8:446. doi: 10.3389/fpubh.2020.00446 (PMC7505925; doi:10.3389/fpubh.2020.00446)
Supplement: Supplementary file 1 [file Table_1.pdf]

Appendix A:

**Steps**	**Tasks**	**Tools**
Step 1 Prepare together	Read the vision of HHR	– Information about the roles within HHR<br>– Explanation about level of dialogue and involvement at each step
	Compose a project team	– Communication tips & fill-in template to create a project group for development
	Develop a project planning	– Information and guidelines for project planning<br>– Information and guidelines for project timeline
	Create commitment and involvement at all levels	– Fill-in HHR poster template<br>– Fill-in HHR presentation template<br>– HHR Flyer
Step 2 Measuring is knowing	Plan, spread, and conduct needs assessment	– Communication tips & checklist to conduct a needs assessment<br>– Checklist privacy<br>– "Healthy at work" Questionnaire
	Analyze results of the needs assessment	– Manual questionnaire analysis<br>– Fill-in report template for results of needs assessment for management
Step 3 Our problems	Communicate the outcomes of needs assessment to employees	– Fill-in presentation template for employees – traffic light model (red - take action; orange - prevent further deterioration; green - maintain)
	Brainstorm about relevant problems other than the results of needs assessment	– Communication tips & working form for brainstorming – post-its
	Prioritize the most important problems	– Communication tips & working form for prioritizing
	Inform all employees about the problem analyses	– Communication tips & fill-in poster template for top 3 problems
Step 4 Our solutions	Identify and review existing solutions (evidence-based)	– Library: two matrices. Matrix 1 with simple solutions. Matrix 2: examples of scientific evidence-based interventions.

		– Overview of useful websites<br>– Checklist to develop an intervention by the organization
	Brainstorm about possible solutions	– Communication tips & working form to conduct ideas about solutions on the work floor<br>– Working form for brainstorming – post-its & brainwriting
	Prioritize the best fitting solutions	– Working form – select top 3 solutions per problem – criteria for prioritizing solutions: feasibility, costs, time, effect.<br>– Working form – formulation of SMART solutions & fill-in template
	Vote by employees on the best solutions	– Fill-in voting cards template for employees to vote on the best solution
	Communicate about the selected solutions	– Communication tips & fill-in poster template for selected solutions
Step 5 Action plan	Decision: How to approach the selected solutions?	– Preparation tips for management meeting & fill-in sheet for preparation and decision document<br>– Letter template for management<br>– Communication tips & checklist for adaptation solutions
	Develop an action plan	– Communication tips & fill-in sheet action plan (W-questions)<br>– Communication tips & fill-in template to create a project group for implementation
	Communicate about the action plan	– Communication tips & fill-in action plan poster template
Step 6 Let's start	Implement the action plan	– Communication tips & checklist implementation
	Periodic evaluation	– Communication tips and approach for evaluation<br>– Working form for evaluation methods
Step 7 Evaluation	Conduct a final evaluation	– Communication tips & approach for evaluation<br>– Working form for evaluation methods
	Plan for sustaining the successful solutions	– Sustainability checklist
Step 8 Along the way: Obstacles in the process		– Tips of do's and don'ts within a dialogue<br>– Working form to improve collaboration
